# Constitutional 763.3 Kb chromosome 1q43 duplication encompassing only CHRM3 gene identified by next generation sequencing (NGS) in a child with intellectual disability

**DOI:** 10.1186/s13039-019-0427-3

**Published:** 2019-04-17

**Authors:** Xiaofei Cheng, Qifang Yang, Jun Liu, Juan Ye, Huiying Xiao, Gaimei Zhang, Yuanyuan Pan, Xia Li, Ruifeng Hao, Yinfeng Li

**Affiliations:** 1 Department of Obstetrics and Gynecology, the First Hospital of Huhhot City, Inner Mongolia, China; 2 Department of Radiology, the First Hospital of Huhhot City, Inner Mongolia, China

**Keywords:** Intellectual disability, 1q43 duplication, CHRM3

## Abstract

**Background:**

Deletion or duplication on the distal portion of the long arm of chromosome 1 result in complex and highly variable clinical phenotype including.

intellectual disability and autism.

**Case presentation:**

We report on a patient with intellectual disability and a 763.3 Kb duplication on 1q43 that includes only CHRM3, which was detected by next generation sequencing (NGS). The patient presented with intellectual disability, developmental delay, autistic behavior, limited or no speech, social withdrawal, self-injurious, feeding difficulties, strabismus, short stature, hand anomalie, and no seizures, anxiety, or mood swings, and clinodactyly.

**Conclusions:**

We propose that CHRM3 is the critical gene responsible for the common characteristics in the cases with 1q43 duplication and deletion.

## Introduction

The clinical phenotype associated with chromosome 1q43-q44 deletion and duplication is characterized by substantial intellectual disability, limited or no speech, and craniofacial dysmorphology, hand and foot anomalies, congenital heart, genital malformation, and central nervous system (CNS) abnormalities, including microcephaly, seizures, and agenesis of the corpus callosum [1]. The clinical phenotype of the terminal region of chromosome 1 deletion and duplication reported by references is highly variable, due to the size of the segment and the number of genes involved [2–11]. Accordingly, qualification and quantification of the critical region/regions associated with these particular characteristics would provide more evidence in genotype-phenotype correlation.

Here, we report the first case of a 763.3 kb duplication at chromosome 1q43 involving only CHRM3 which encodes for the M3-muscarinic receptor, who has intellectual disability, developmental delay, autistic behavior, short stature, strabismus, hand anomalies, limited or no speech, feeding difficulties, social withdrawal, self-injurious, and a tendency toward self-injurious stereotypic behavior. The clinical features of this patient with pure 1q43 duplication are different from a previously reported case of a ~ 3½ year-old boy with a pure 473 kb deletion at 1q43 comprising only CHRM3, who has no short stature and hand anomalies [12]. We propose that CHRM3 is the critical gene responsible for the common characteristics in the cases with 1q43 duplication and deletion.

## Materials and methods

### Case presentation

**Fig. 1:**
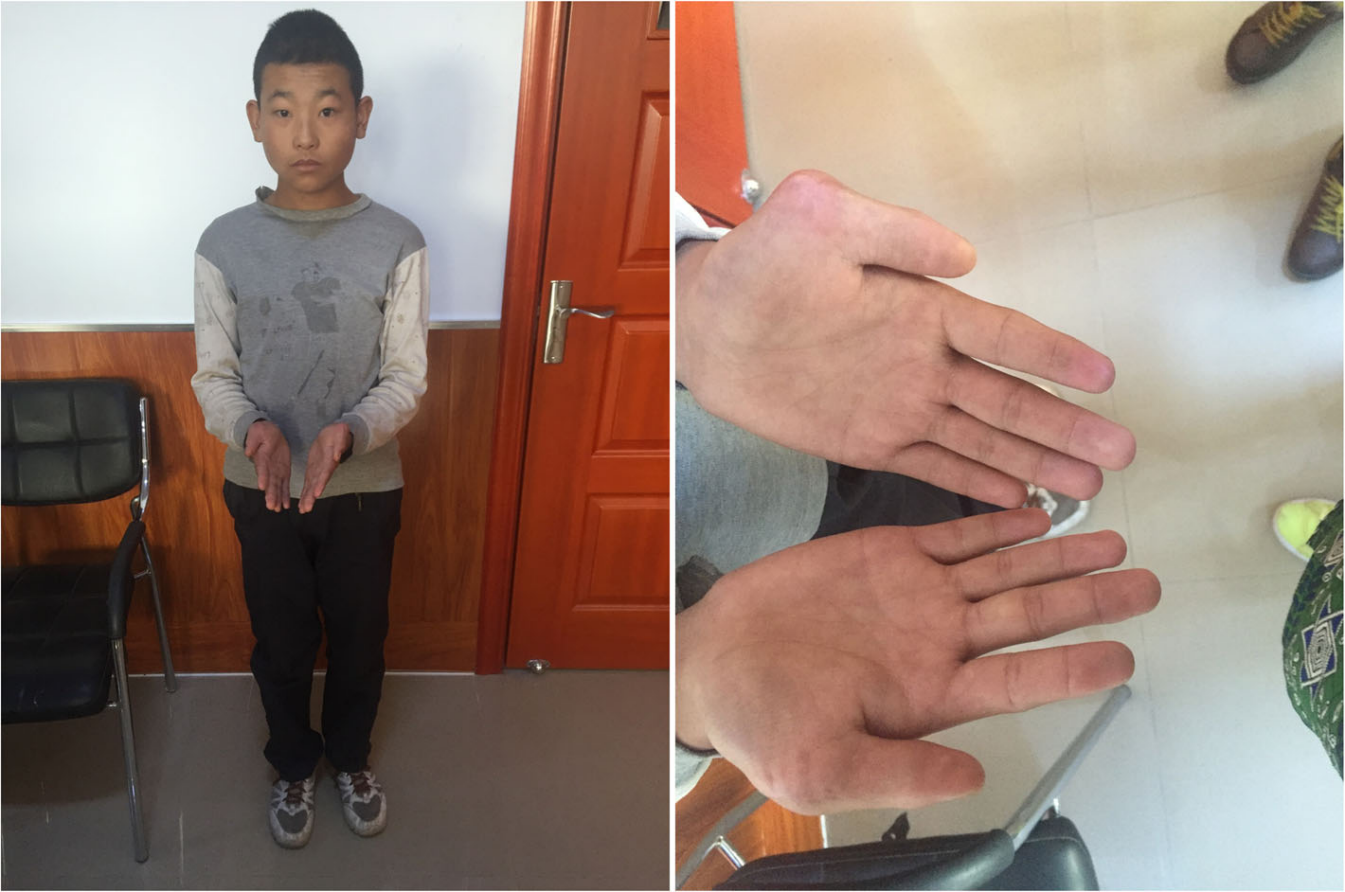
The figure shows some clinical features like strabismus, short stature, and hand anomalie

The patient, a boy, was born at term via uncomplicated spontaneous vaginal delivery to an 24-year-old gravida at 38 weeks of gestation. His birth weight was 3.5 kg. Prenatal course had no preeclampsia; neonatal history was benign. Both parents had no history of neurological disease and developmental delays. At ~ 12 months, his parents became concerned for delays in language skills. At 3 yrs., he was given a diagnosis of autism disorder by pediatric evaluation. His past medical history is significant for strabismus, short stature and hand anomalie (Fig. 1). He is impulsive, hyperactive and inattentive in terms of behavior, and has severely limited social skills.

### Next generation sequencing (NGS) analysis

DNA was extracted from peripheral blood at 2018 January using QIAamp DNA Blood Mini Kit (Qiagen, Germany), and was quantified by using Qubit dsDNA BR Assay Kit and Qubit 2.0 Fluorometer (Life Technologies, USA). KAPA HTP Library Preparation Kit Illumina® Platforms (Kapa Biosystems, USA) was utilized for highthroughput library construction for Illumina sequencing, starting from fragmented, double-stranded DNA (dsDNA) according to manufacturer’s instructions. Reads of the sequencing run for patient, patient’s father and mother were 8.5 M, 9.2 M, 9.8 M, respectively. After quality control, coverage percentages of ROI were calculated using the filtered reads at 0.20X, 0.22X, 0.23X for patient, patient’s father and mother, respectively. Genome Analyzer Toolkit (GATK) was used for base quality score recalibration, and single-nucleotide variants (SNV) discovery across was performed for all three samples. The whole-exome enrichment and sequencing (WES) was determined based on the Agilent SureSelect Human All Exon platform (Agilent Technologies), which targets ~ 50 Mb of the human exonic regions. The SureSelect system uses ~ 120-base RNA probes to capture known coding DNA sequences (CDS) from the NCBI Consensus CDS Database as well as other major RNA coding sequence databases, such as Sanger miRBase.

## Results

**Fig. 2:**
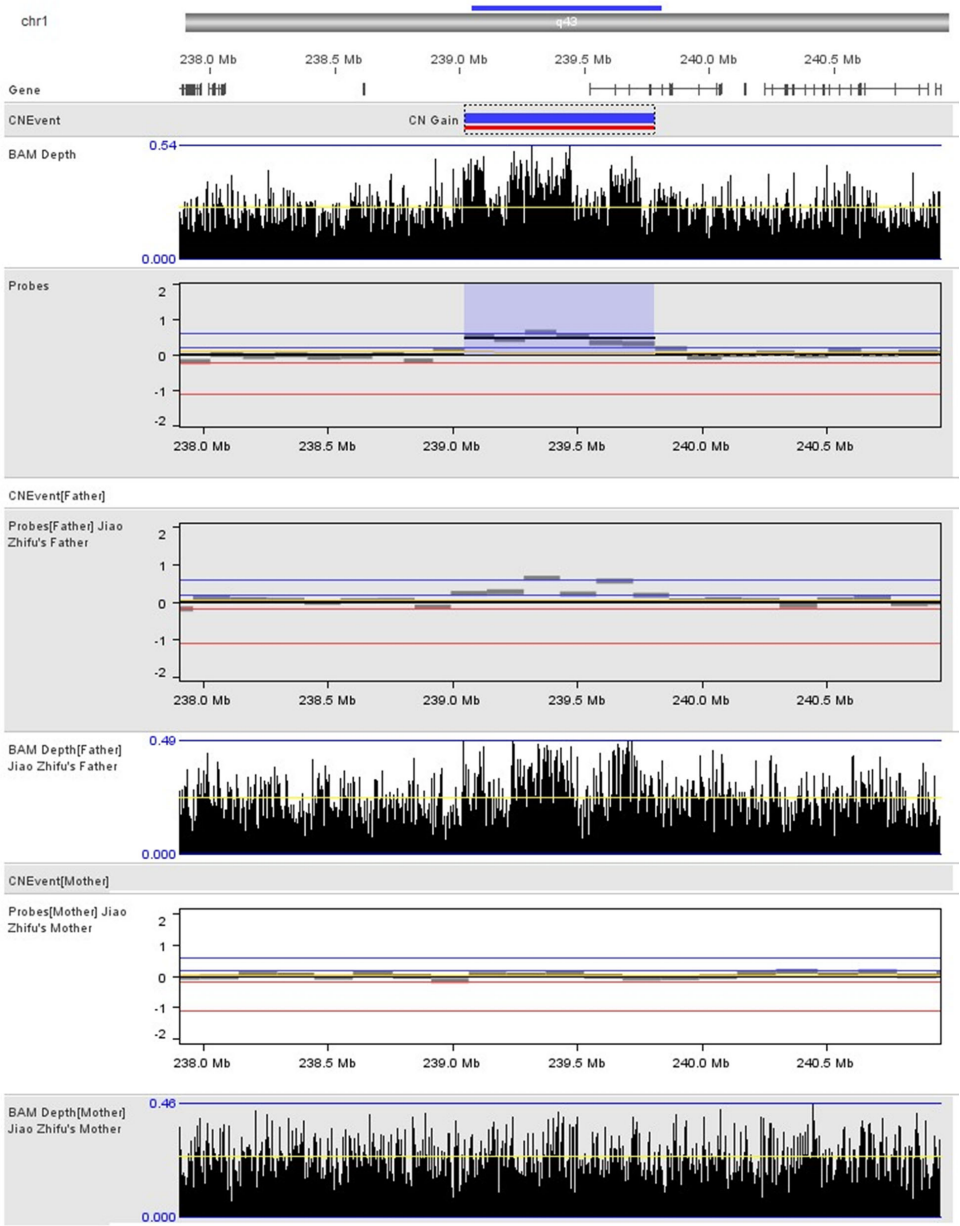
Overview of chromosome 1 with a 763.3 kb duplication of 1q43 region involving CHRM3. The region of interest has been magnified. The inheritance of duplication in our case could not be observed in parental samples

**Fig. 3:**
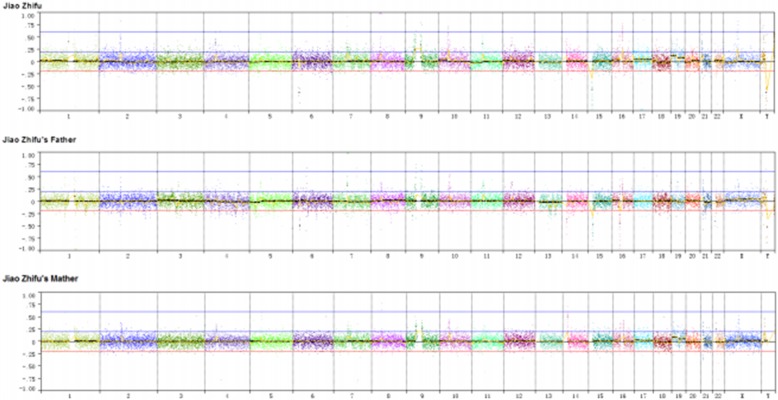
Genome maps of patient, patient’s father and mother

**Fig. 4:**
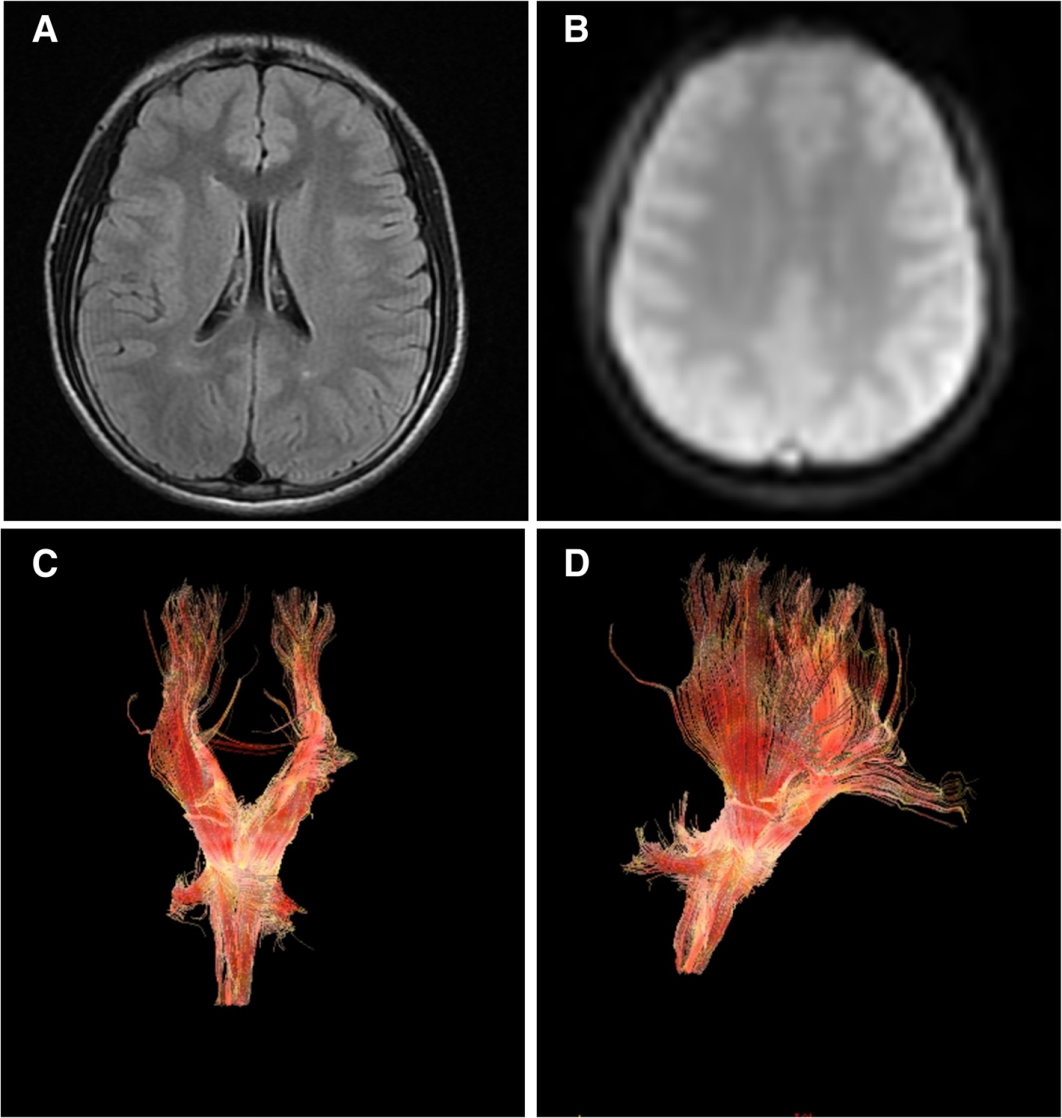
MRI of the head showed normal structures. **a** Axial FLAIR image at the level of the lateral ventricles. **b** Diffusion Tensor Imaging (DTI) image. **c**, **d** DTI color maps

**Fig. 5:**
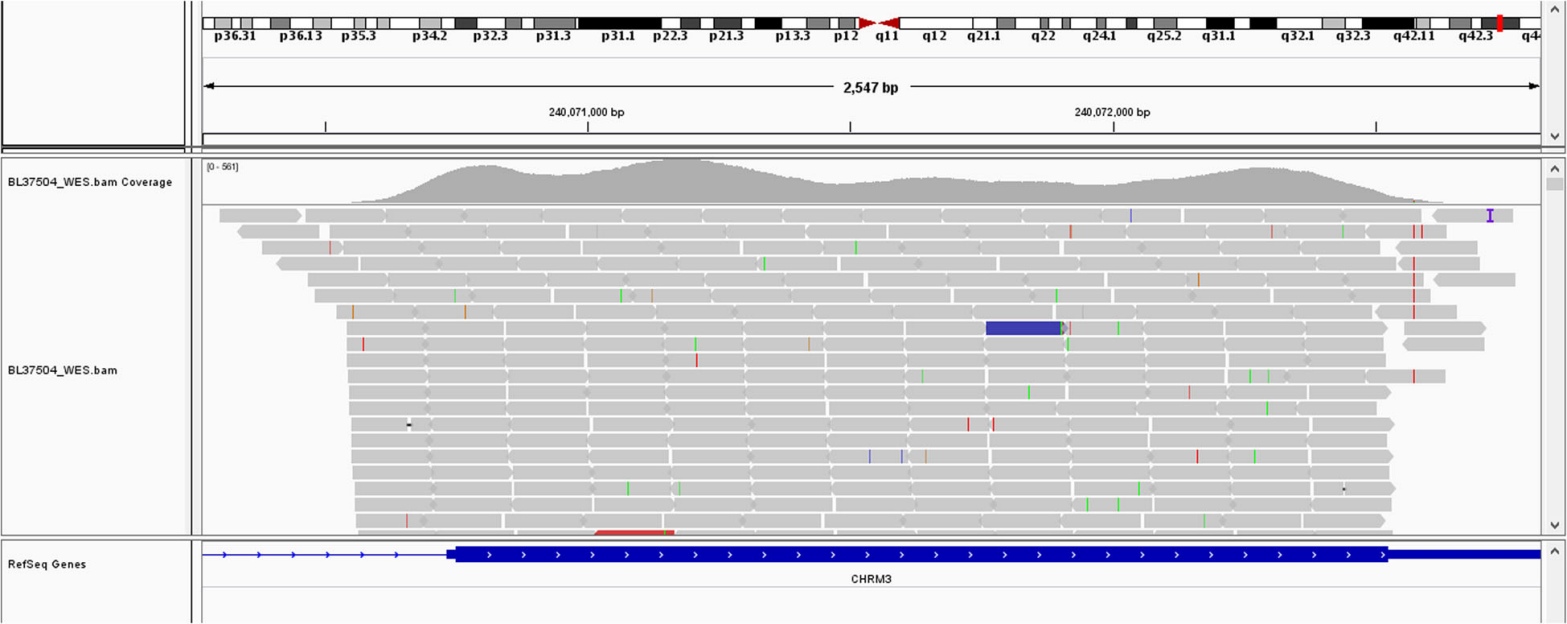
Whole-exome sequencing. The single-nucleotide polymorphism (SNPs) were not found in CHRM3

As a part of intellectual disability and autism workup, the patient undergoes genetic testing. NGS analysis showed a single duplication of 763.3 Kb at 1q43 region (239,049,010-239,812,310, GRCh37, NCBI) (Figs. 2 and 3), including only CHRM3. The schematic of chromosome 1q43 region is shown in Fig. 2. The clinical manifestations of the case with a pure 473 kb deletion at 1q43 comprising only CHRM3 reported by Petersen et al. [12], along with the present case are presented in Table 1. The inheritance of duplication in our case could not be observed in parental samples. MR images of the patient’s head showed normal structures (Fig. 4). CHRM3 gene was determined using whole-exome enrichment and sequencing. And the single-nucleotide polymorphism (SNPs) were not found in CHRM3 (Fig. 5).

**Table 1:** Features presented in patients with 1q43 deletion and duplication

Features	Patient reported by Petersen et al. [12]	Patient in this report
Age, Gender	3 Years 7 Months, Male	14 Years, Male
Coordinates	239,412,391e239,885,394	239,049,010-239,812,310
Cytoband	1q43	1q43
Size	473 kb	763.3Kb
Important genes	CHRM3	CHRM3
Origin	NA	–
Microcephaly	–	–
Intellectual disability	+	+
Developmental delay	+	+
Autistic behavior	+	+
Seizure	–	–
Feeding difficulties	+	+
Short stature	–	+
Clinodactyly	–	–
Strabismus	+	+
Self-injurious	+	+
hand anomalie	–	+
Brain MRI	Normal	Normal

## Discussion

We report the first case of a 763.3 kb duplication at chromosome 1q43 involving only CHRM3 (Acetylcholine receptor, muscarinic, 3; OMIM: 118494) which encodes for the M3-muscarinic acetylcholine receptor. M3-muscarinic acetylcholine receptor is the predominant muscarinic subtype mediating acetylcholine-induced airway smooth muscle contraction, one of 5 subtypes (M1-M5) of muscarinic receptors, found throughout the CNS and peripheral tissue. The clinical features of this patient with pure 1q43 duplication are different from a previously reported case of a ~ 3½ year-old boy with a pure 473 kb deletion at 1q43 comprising only CHRM3 [12] (Table 1). Both children exhibit substantial intellectual disability, developmental delay, autistic behavior, limited or no speech, social withdrawal, self-injurious, feeding difficulties, strabismus, and no seizures, anxiety, mood swings, and clinodactyly. However, the patient in our report has short stature and hand anomalie, which were not observed in the patient with 473 kb deletion including CHRM3 reported by Petersen et al. [12]. The deletion in the patient reported by Petersen et al. includes a 380 kb upstream region and the first four exons of CHRM3, whereas in our patient with a 763.3 kb duplication at 1q43 includes 343 kb in CHRM3 which involves the first seven exons of CHRM3 [13]. The entire cDNA encoding the m3 receptor comprise 4559 bp in 8 exons, exon 1 includes a cluster of transcriptional start sites, exons 2, 4, 6 and 7 are subject to alternative splicing [13]. The difference in clinical features might because of the various length of CHRM3 gene involved. As short stature was observed in a previously reported case of a 7 year-old autistc boy with a 911 kb deletion at 1q43 encompassing all 8 exons of CHRM3 and two other genes: RPS7P5 and FMN2 [14]. Moreover, compared with 1q43-q44 deletion, hand abnormalities appear to be more associated with 1q43-q44 duplication [15–23]. However, abnormal hand was not observed in a report that a deletion and a duplication of approximately 6.0 Mb at 1q43-q44 involving CHRM3 in the proband and her younger brother [15]. Compared with 1q43-q44 deletion, the clinical manifestations of the patient with 1q43-q44 duplication appear to be mild and mainly include macrocephaly, mental retardation and mild malformation.

The muscarinic acetylcholine receptors belong to the family of G protein-coupled receptors that regulate numerous fundamental functions of the central and peripheral nervous systems. The muscarinic acetylcholine receptors consist of five distinct subtypes (M1, M2, M3, M4, M5) which encoded by the genes CHRM1, CHRM2, CHRM3, CHRM4 and CHRM5. M1, M3 and M5 receptors tend to couple to G proteins of the Gq/11 family, whereas M2 and M4 receptors preferentially signal through the Gi/o family of G proteins [24]. The M3 muscarinic acetylcholine receptor (M3R) is distributed dominantly in the central and peripheral nervous systems, cardiac muscles, smooth muscles, and exocrine glands, plays a central role in many fundamental functions of the CNS and other human physiology, including regulating heart rate, smooth muscle contraction, and glandular secretion [25]. In CNS, M3-muscarinic receptors have been specifically related to behavior modulation, for example, M3-muscarinic receptor activation could stimulation dopamine release and enhances signal transduction in the hippocampus (related to memory acquisition), striatal nucleus (related to reward and conditioning), nucleus accumbens (related to addiction and pleasure) and amygdala (related to fear conditioning and risk taking). The KO mice for the CHRM3 gene were found deficits in tasks involving fear conditioning, learning and memory [26]. Furthermore, CHRM3 expression was different in the 12 different brain regions. The brain stem, cerebellum, and olfactory region showed decreased expression patterns. As 8 exons of CHRM3 gene were derived from three distinct promoter regions (T1: L1HS, T2, T4: original, T3, T3–1:THE1C) [27], these transposable element-derived transcripts showed tissue-specific expression patterns. One retroelement could be cleaved by other retroelement integration events in different tissues. Thus, the various exons of CHRM3 gene involved might account for the differences in clinical features between our patient with 1q43 duplication and the case with 1q43 deletion. Much about the specific role of CHRM3 in human CNS pathophysiology remains to be elucidated, identification of well-detailed genotype-phenotype correlations in this case as well as others of 1q43 duplication and deletion would provide more possible links between M3-muscarinic receptor pathophysiology and the neurocognitive phenotype. For example, it was reported that SNPs rs6700381 in CHRM3 gene in 1q43 was associate with its role in multiple brain functional connectivities [28]. In this case, SNPs were not found in CHRM3. Therefore, a duplication of 763.3 Kb at 1q43 region might result in abnormal protein structure of CHRM3, which might account for the clinical features.

## Conclusions

We report the first case of a 763.3 kb duplication at chromosome 1q43 involving only CHRM3. In view of these common characteristics in our patient and the case with 1q43 deletion, we propose CHRM3 as the critical gene responsible for intellectual disability, developmental delay, autistic behavior, limited or no speech, social withdrawal, self-injurious, feeding difficulties, strabismus, and other overlapping phenotype in our patient as well as other patients with 1q43 deletions and duplication including CHRM3.

## Abbreviations

CNS: Central nervous system
M3R: M3 muscarinic acetylcholine receptor
NGS: Next generation sequencing
